# Hydration of protein–RNA recognition sites

**DOI:** 10.1093/nar/gku679

**Published:** 2014-08-11

**Authors:** Amita Barik, Ranjit Prasad Bahadur

**Affiliations:** Department of Biotechnology, Indian Institute of Technology Kharagpur, Kharagpur-721302, India

## Abstract

We investigate the role of water molecules in 89 protein–RNA complexes taken from the Protein Data Bank. Those with tRNA and single-stranded RNA are less hydrated than with duplex or ribosomal proteins. Protein–RNA interfaces are hydrated less than protein–DNA interfaces, but more than protein–protein interfaces. Majority of the waters at protein–RNA interfaces makes multiple H-bonds; however, a fraction do not make any. Those making H-bonds have preferences for the polar groups of RNA than its partner protein. The spatial distribution of waters makes interfaces with ribosomal proteins and single-stranded RNA relatively ‘dry’ than interfaces with tRNA and duplex RNA. In contrast to protein–DNA interfaces, mainly due to the presence of the 2′OH, the ribose in protein–RNA interfaces is hydrated more than the phosphate or the bases. The minor groove in protein–RNA interfaces is hydrated more than the major groove, while in protein–DNA interfaces it is reverse. The strands make the highest number of water-mediated H-bonds per unit interface area followed by the helices and the non-regular structures. The preserved waters at protein–RNA interfaces make higher number of H-bonds than the other waters. Preserved waters contribute toward the affinity in protein–RNA recognition and should be carefully treated while engineering protein–RNA interfaces.

## INTRODUCTION

Water molecules are ubiquitous in living cells. They are responsible for the three-dimensional structural integrity and functions of biomolecules by participating in all physical and chemical interactions with them. Their integral role in regulating thermodynamics and kinetics of biomolecules governing different cellular processes is well studied (1–8). They also contribute significantly in enthalpy and entropy of free energy required for the folding and the binding of biomolecules (9–14). The importance of the role of water molecules at the macromolecular binding interfaces was first emphasized by Otwinowski *et al*. (15) in the tryptophan repressor-operator complexes. Later, Bhat *et al*. (16) showed that several of the ordered water molecules in the free antibody combining site are retained and that additional water molecules link antigen and antibody upon complex formation. Subsequently, many studies confirmed the role of water molecules in the stability of the macromolecules and their complexes. A water molecule can act as both hydrogen bond (H-bond) donor and acceptor, and can take part in multiple H-bonds. A water molecule can bridge the binding interfaces by making H-bonds with both the interacting partners (17,18) or it can be buried in the monomer structure stabilizing its tertiary fold (19). Moreover, water molecules can act as a buffer to screen unfavorable electrostatic interactions at the protein–nucleic acid interfaces (8,20). Barillari *et al*. (21) classified water molecules into two classes: those conserved and not displaced by the ligands, and those that can be displaced by the ligands. They found that the conserved water molecules have higher coordination number of H-bonds and tightly bound than the displaced water molecules. Depending upon the relative location of the hydration waters, Nakasako (22) divided them into four classes: ‘inside’, ‘contact’, ‘first-layer’ and ‘second-layer’. The ‘inside’ waters are located at the protein cavities, and have approximately four H-bond partners. The ‘contact’ waters mediate intermolecular interactions, and have three to four H-bond partners. Water molecules in the ‘first’ layer directly interact with the atoms of the protein surface through H-bonds and/or van der Waals contacts. Molecules in the ‘second’ layer have no direct interaction with protein. In another work, Li *et al.* (23) classified water molecules on the basis of their interactions with the protein and RNA and their positional relationship with bulk water. They defined single-water bridge as a water molecule forming H-bonds with both protein and RNA, double-water bridge as a water molecule with H-bonds to one molecule (protein or RNA) of the interface and to another water that is in turn H-bonded to the other molecule (RNA or protein) and single or double hydrophobic bridge as a water molecule in contact with the hydrophobic surfaces at the interface. This classification has been further used to predict the location of interfacial water molecules in protein–RNA interfaces. Although, in recent past, the role of water molecules in protein–protein and protein–DNA recognition has been extensively studied (24–33), an understanding about their role in protein–RNA recognition is still elusive (34–36). The growing number of high-resolution X-ray structures of protein–RNA complexes as well as the free form of their binding partners in the Protein Data Bank (PDB) (37) impelled us to investigate the role of water molecules in protein–RNA recognition.

In this work, we have thoroughly curated a dataset of 89 protein–RNA complexes with bound crystallographic waters from the PDB. We have identified the waters at the interfaces of these complexes. Their role in the recognition process has been comprehensively analyzed by quantifying their number at each interface, and the types of interactions they make with the amino acid residues and the nucleotides. In addition, we have investigated their spatial distribution at the recognition sites, and their specificity to bind with the protein secondary structures as well as the major and the minor grooves of the RNA. Moreover, we have identified the preserved interface waters and specified their role in the recognition process by analyzing the bound and the unbound structures of the components involved in the complex formation. We have extended our analysis to binary protein–DNA and protein–protein complexes, and compared our findings among these three cases to decipher the role of water molecules in molecular recognition in each case.

We find that the hydration of protein–RNA interfaces differs from the hydration of protein–DNA and protein–protein interfaces in terms of the number of interface water molecules as well as the water-mediated H-bond density. About one-third of the interface waters bridge the protein–RNA interface by making H-bonds with the polar groups on both sides. Majority of the interface waters are involved in multiple H-bonds. However, a fraction of them do not make any H-bond with either side of the interface. They may contribute to the van der Waals interaction. Moreover, those making H-bonds have preferences to interact with RNA than its partner protein. We find the chemical groups at the protein–RNA interface have preferences to make H-bonds with the water molecules: the neutral polar groups of proteins are preferred than the charged groups; the ribose of RNA is preferred than the phosphate or the bases. This trend is different in protein–DNA interfaces, where the phosphate is preferred than the bases, while the sugar plays insignificant role. Our analysis on the spatial distribution of the interface waters shows that the interfaces with tRNA and duplex RNA are relatively ‘wet’ than the interfaces with ribosomal proteins and single-stranded RNA. We find that the strands at the protein–RNA interfaces make higher number of water-mediated H-bonds per unit interface area than the helices or the non-regular secondary structural elements. Additionally, in contrast to protein–DNA interfaces where the major groove of DNA is hydrated more than its minor groove, the minor groove of RNA is hydrated more than its major groove in interfaces involving duplex RNA. Our analysis reveals that the preserved waters make higher number of H-bonds compared to the other waters at the protein–RNA interfaces. One should be careful about displacing the preserved waters while engineering a protein–RNA interface.

## MATERIALS AND METHODS

### Dataset of protein–RNA complexes with bound crystallographic water molecules

We curated the PDB for binary protein–RNA complexes with resolution 2.6 Å or better, and left out permanent multisubunit assemblies such as ribosomes and viral capsids for a separate study. All the complexes in the dataset are non-obligate with the possible exception of complexes with ribosomal proteins. We selected 480 structures with polypeptide chains of minimum 30 amino acid residues, and polyribonucleotides of minimum five nucleotides. In order to remove the redundancy, we performed pairwise sequence alignment for all the entries in the dataset using BLAST (http://blast.ncbi.nlm.nih.gov/Blast.cgi). When the protein components in two entries had more than 35% sequence identity, only the one with better resolution was kept. Additionally, we kept the complexes with homologous protein chains if they bind different RNA sequences, otherwise removing one of them would lose the result of diversity of interactions shown by them. We also performed the pairwise sequence alignment using structural superposition in PDBeFold (http://www.ebi.ac.uk/msd-srv/ssm/cgi-bin/ssmserver). The sequence identity values obtained using structural superposition are essentially similar to those obtained using BLAST. Finally, we retained 89 complexes reporting at least one interface water molecule (Supplementary Table S1). Water molecules within a distance of 4.5 Å from at least one of the interface atoms of both the interacting partners were identified at the interface. We have used shorter (or longer) cut-off distances (3.0–5.0 Å) for selecting the interface waters. This gave us somewhat less (or more) interface waters (Supplementary Figure S1), but did not alter the general conclusions that we obtained using 4.5 Å. When the asymmetric unit contains multiple copies of the complex, only one copy was kept. In case of oligomeric structures, the data corresponding to the biologically significant oligomeric state were selected after verification from the literature and the PISA server (38). We carefully checked each and every structure for modified residues and nucleotide bases, which are marked with the keyword ‘HETATM’ in the coordinate list in a PDB file. We kept them as their corresponding amino acids and bases by changing the keyword ‘HETATM’ to ‘ATOM’, a list of which is given in the Supplementary Table S2. According to Bahadur *et al.* (36), the dataset was divided into four classes: (A) complexes with tRNA, (B) complexes with ribosomal proteins, (C) complexes with duplex RNA and (D) complexes with single-stranded RNA.

### Interface area, preserved water molecules and hydration pattern

The size of a protein–RNA interface was estimated by measuring the solvent accessible surface area (ASA) buried in contact. The web server PRince (39) was used for calculating interface area (B), which was estimated by subtracting the ASA of the complex from the sum of the ASAs of the individual subunits. ASA values were calculated using the program NACCESS (40), which implements the Lee and Richards (41) algorithm, using a probe radius of 1.4 Å and default group radii. H-bonds were identified with the program HBPLUS (42) using the default parameters. The program DSSP (43) was used for the secondary structure assignment to protein chains.

The unbound structures of protein and RNA components of a complex were obtained by performing BLAST (44) search for each of the sequence against the non-redundant PDB with sequence identity >90%, query coverage of >95% and an *E*-value <10^−3^. When multiple candidates for an unbound structure were found, entry with the highest sequence similarity and the maximum alignment length with the bound structure was kept. While selecting the unbound structures, we kept the same resolution limit as that of the bound structures. It was further confirmed that the unbound forms corresponding to the bound structures contained water molecules. This finally gave us 38 unbound proteins and seven unbound RNAs for the complexes listed in Supplementary Table S1. An interface water was considered preserved if it makes an H-bond with the same donor or acceptor atoms of protein or RNA chains in both the bound and unbound structures. The dataset of protein-DNA and protein-protein complexes are taken from Setny *et al*. (45) and Hwang *et al*. (46), respectively.

The structural parameter *d*_r_ was used to quantitatively define a ‘wet’ or a ‘dry’ protein–RNA interface. Briefly, *d*_r_ is the ratio of the average distance of interface waters to the average distance of interface atoms contributed by both protein and RNA chains. In both the cases, distances were measured from the center of mass of the interface. Interfaces having *d*_r_ value less than or equal to 1 were designated as ‘wet’, and otherwise as ‘dry’.

## RESULTS

### The complexes with interface water molecules

**Figure 1.**
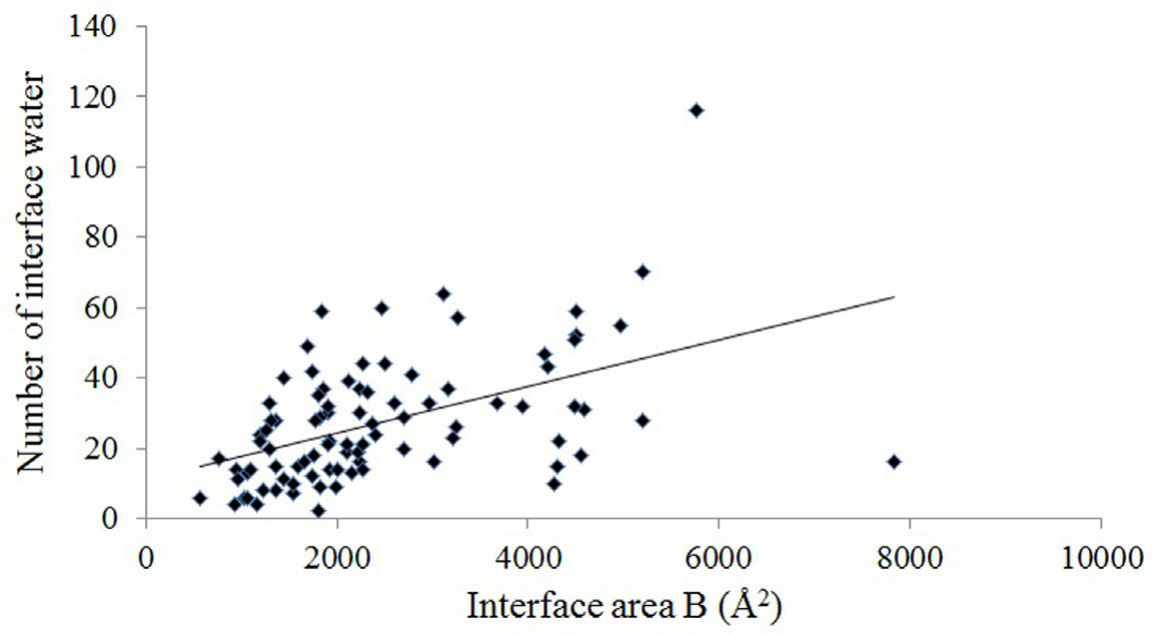
The number of interface water molecules is plotted against the interface area B in protein–RNA complexes.

The dataset consists of 89 X-ray structures of protein–RNA complexes with resolution 2.6 Å or better (Supplementary Table S1), reporting a total of 19 327 crystallographic waters bound to them. We have identified 2440 waters (∼13% of all the bound crystallographic waters) bound at the interface, contributing to the recognition process. Thus, an average protein–RNA complex contains about 217 bound waters, of which, 27 (equivalent to 11 per 1000 Å^2^ of B) are at the interface (Table 1). The number of interface waters ranges from 4 to 116, and tends to increase with B (Figure 1), although the correlation between them is mediocre (Pearson correlation coefficient *R =* 0.49). Exceptionally, the interface between RNA-dependent RNA polymerase of bacteriophage phi6 and a 6 nt RNA (PDB id: 1UVI) contains only two waters. Here, the small RNA molecule interacts with the core region of the polymerase, which is almost dehydrated (47). On the other hand, the interface between arginyl-tRNA synthetase and its cognate tRNA (Arg) (PDB id: 1F7U) contains 116 waters, highest in this dataset. This complex contains 588 bound waters, and it has been observed that a large number of water-mediated interactions confer a high adaptability to the interface while providing the required specificity and affinity in the recognition process (48). The wide range of the number of interface waters may be attributed to the inconsistent representation of the solvent position in the crystallographic studies, which is evident by the presence of fewer molecules in low-resolution X-ray structures (18). However, this range remains wide, 4–64, in a 1.8 Å subset, suggesting strongly that in spite of the inconsistent reporting there are real differences between protein–RNA interfaces in terms of hydration. On an average, interfaces with tRNA are more than twice larger than those with ribosomal proteins and single-stranded RNA (Table 1). Although the interfaces with tRNA contain the highest number of water molecules, their surface density is less due to the large size of their interfaces. Among the different classes, interfaces with tRNA are less hydrated (9.3 water molecules per 1000 Å^2^ of B) compared to interfaces with single-stranded RNA (11.0 per 1000 Å^2^ of B) or duplex RNA (12.4 per 1000 Å^2^ of B). In contrast, interfaces with ribosomal proteins are most hydrated (14.5 per 1000 Å^2^ of B). This lowest density of immobilized waters at the tRNA interfaces is justified by their relatively more hydrophobic nature compared to the other three classes (Table 1). ANOVA (analysis of variance) test was performed to find the statistical significance in the differences in mean number of interface waters of four classes. The *P*-value (at *α* = 0.05 level of significance) obtained is 0.0091, rejecting the null hypothesis (mean values are same in all the four classes).

**Table 1.** Statistics of interface water molecules

Interface statistics	Protein–RNA					Protein–DNA^a^	Protein–protein^b^
	tRNA	ribosomal	duplex	single-stranded	All classes		
Number of complexes	12	5	27	45	89	115	115
Interface area B (Å^2^)	4185 ± 1226	1725 ± 434	2589 ± 1052	2004 ± 1219	2460 ± 1347	3137 ± 1350	1886 ± 704
Non-polar area fraction *f*_np_^c^ (%)							
Protein	56 ± 3	52 ± 11	54 ± 8	57 ± 8	56 ± 8	54 ± 7	60 ± 6
RNA	35 ± 2	29 ± 7	32 ± 5	34 ± 6	33 ± 6	37 ± 5	-
Mean number of bound water^d^	301 ± 195	153 ± 59	208 ± 108	208 ± 122	217 ± 131	232 ± 174	218 ± 159
Interface water^e^							
Range	8–116	12–49	9–70	2–51	2–116	2–174	1–58
Mean number per interface	39 ± 29	25 ± 15	32 ± 18	22 ± 12	27 ± 18	44 ± 32	20 ± 14
Per 1000 Å^2^ of B	9.3	14.5	12.4	11.0	11.0	14.0	10.6
Water-mediated H-bonds^f^							
Range	16–198	23–78	14–134	5–130	5–198	2–244	0–101
Mean number per interface	76 ± 50	45 ± 22	60 ± 34	41 ± 26	52 ± 34	70 ± 47	31 ± 22
Per 1000 Å^2^ of B	18.2	26.1	23.2	20.5	21.1	22.3	16.4
Bridging water molecules^g^							
Range	3–34	4–12	2–23	0–26	0–34	0–60	0–15
Mean number per interface	15 ± 9	8 ± 3	12 ± 7	8 ± 5	10 ± 7	15 ± 11	5 ± 3
Per 1000 Å^2^ of B	3.6	4.6	4.6	4.0	4.1	4.8	2.7
Preserved water molecules^h^							
Range	1–12	4–6	3–14	0–17	0–17	-	-
Mean number per interface	4	5	7	6	6	-	-
Per 1000 Å^2^ of B	2.2	5.8	4.7	6.8	5.2	-	-
Hydration pattern							
Mean *d*_r_	0.86 ± 0.09	1.17 ± 0.05	0.98 ± 0.12	1.07 ± 0.17	1.02 ± 0.16	1.01 ± 0.14	1.06 ± 0.16

^a^Parameters are calculated on a protein–DNA dataset taken from (45) with resolution 2.6 Å or better, and having at least one interface water molecule.

^b^Parameters are calculated on a dataset of protein–protein complexes taken from the docking benchmark 4.0 (46) with resolution 2.6 Å or better, and having at least one interface water molecule.

^c^Percentage of area contributed by the non-polar groups (all carbon-containing groups).

^d^All the crystallographic water molecules bound to the complex.

^e^Water molecules <4.5 Å from atoms of both protein and RNA chains.

^f^H-bonds are calculated using the program HBPLUS (42).

^g^Water molecules making H-bonds with the polar groups of both protein and RNA chains.

^h^At least one water-mediated H-bond with the same atom of either protein or RNA chain is found in both bound and unbound form. The mean number and the density are calculated over all the preserved water molecules in the dataset.

### Water-mediated polar interactions

Polar interactions at the protein–RNA interface are quantified in terms of H-bonds. The dataset of 89 complexes contains 2440 interface waters, of which 442 (∼18%) do not make any H-bond with the polar groups of the binding partners. The remaining 1998 waters make 4625 H-bonds with a biased distribution: 58% with the RNA polar groups and 42% with the protein polar groups. This asymmetry is also observed in interface area, where the RNA side contributes more compared to the protein side (31). Of the 442 interface waters, 367 (83%) make at least one H-bond with the other interface waters, while the rest, 75 (only 3% of total interface water), do not participate in any H-bond. The average distance of these non-H-bonded waters from the closest interface atom is 3.5 Å (a distribution is given in Supplementary Figure S2), and we find some of them are trapped at the interface cavities (Supplementary Figure S3). This suggests that these non-H-bonded waters may contribute to the van der Waals interactions (49,50). On an average, a protein–RNA interface is stabilized by 52 water-mediated H-bonds, in addition to 20 direct H-bonds between the polar groups of protein and RNA. This indicates that each water molecule makes about 1.9 H-bonds, which corresponds to one per 47 Å^2^ of B. Thus, the frequency of water-mediated H-bonds is almost 2.7 times higher than the direct protein–RNA H-bonds, which is one per 125 Å^2^ of B (36). Table 1 shows the variation of water-mediated H-bond density in different classes. While the class with ribosomal proteins has the highest density, one per 38 Å^2^ of B, the class with tRNA has the lowest density, one per 55 Å^2^ of B. The classes with duplex RNA and single-stranded RNA have intermediate density, one per 43 and 49 Å^2^ of B, respectively. The *P*-value (at *α* = 0.05 level of significance) obtained from ANOVA test on mean number of water-mediated H-bonds is 0.0052, showing the significant statistical difference in mean values among the four classes.

More than one-third (36%) of the interface waters make at least one H-bond with the polar groups on both sides of a protein–RNA interface. They are defined as bridging waters. Table 1 shows that an average protein–RNA interface contains 10 bridging waters, which corresponds to 4.1 waters per 1000 Å^2^ of B. The density of bridging waters varies in different classes; it is lower in interfaces involving tRNA and single-stranded RNA, and higher in interfaces involving ribosomal proteins and duplex RNA. The *P*-value (at *α* = 0.05 level of significance) for mean number of bridging waters among the four classes is 0.0088, confirming the significance of statistical differences.

### Water involving multiple H-bonds

**Figure 2.**
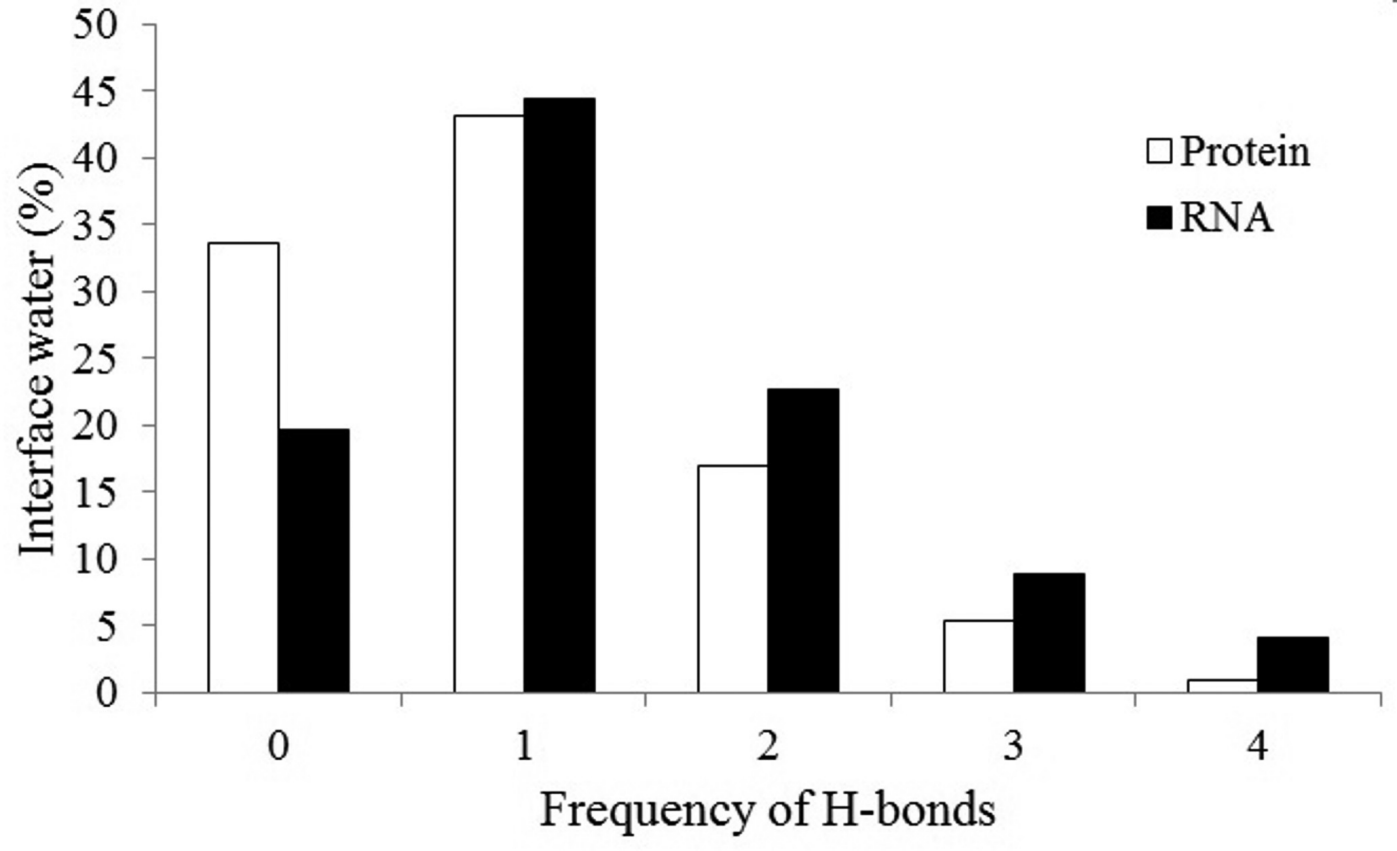
Number of interface water molecules making zero to four H-bonds to the protein or the RNA component. The bars under ‘0’ H-bond represent the number of water molecules making no H-bond with one partner, but they make H-bonds with the other partner.

Bridging waters must make at least one H-bond with both the interacting partners. Since water has two donors and two acceptors, tetrahedrally oriented, just two H-bonds will not be specific because the water can potentially rotate to present either a donor or acceptor to the base (27). Therefore, specificity only arises when the water makes more than two H-bonds simultaneously. Figure 2 shows the frequency distribution of interface waters making up to four H-bonds. About 60% of them make one or two H-bonds with the protein polar groups. This percentage increases to 67% with the RNA polar groups. These waters are involved in non-specific recognition, mostly filling up the interface cavities. They also contribute toward maximizing the surface complementarity at the interface (26). Besides, only 6% of the interface waters make three or four H-bonds with the protein polar groups. This percentage increases to 13% with the RNA polar groups. They largely contribute to the specificity in the recognition process, which is exemplified in the interface of arginyl-tRNA synthetase and its cognate tRNA (Arg) (PDB id: 1F7U) (48). This interface contains 116 waters, and 96 of them make at least one H-bond with an interface atom. Delagoutte *et al.* (48) hypothesized that the water-mediated interactions confer a high adaptability to the interface while providing the required specificity and affinity. We identified a significant number (20%) of waters making three and four H-bonds at this interface, contributing to the specificity of the recognition. In our dataset, 34% of interface waters do not make any H-bond with the protein polar groups, but they make H-bonds with the RNA polar groups. Counter example also exists, however, with a lower fraction of 20%, indicating that waters at the protein–RNA interfaces have preference to make H-bonds with RNA than its partner protein.

Table 2 reports the average number of water molecules making multiple H-bonds in each interface in different classes. It shows that the frequency of water molecules making one or two H-bonds is higher than those making three or four H-bonds in the entire dataset, as well as in four different classes. However, the number of such waters differs across the different classes. We find about 18% of interface waters do not make any H-bond. This percentage varies from 16% in interfaces with tRNA and single-stranded RNA to 20% in interfaces with ribosomal proteins and duplex RNA.

**Table 2.** Frequency of interface water making multiple H-bonds

Complexes	tRNA		ribosomal		duplex		single-stranded		All classes	
	Protein	RNA	Protein	RNA	Protein	RNA	Protein	RNA	Protein	RNA
H-bond										
Frequency^a^										
Zero^b^	11.4	6.3	9.0	3.0	9.2	4.6	5.4	4.0	7.6	4.4
One	14.9	13.2	7.4	8.2	11.4	12.0	7.5	8.1	9.7	10.0
Two	5.3	8.6	2.8	5.0	4.0	5.1	3.4	4.2	3.8	5.1
Three	1.3	3.7	0.6	2.0	1.1	2.9	1.3	1.0	1.2	2.0
Four	0.1	1.2	0.0	1.6	0.3	1.3	0.2	0.6	0.2	0.9

^a^Average number of interface water molecules making up to four H-bonds per interface. Total 1998 interface water molecules making at least one H-bond.

^b^Frequency of interface waters that do not make any H-bond with the protein, but they make H-bonds with the RNA and vice versa.

### Chemical groups involving polar interactions

**Figure 3.**
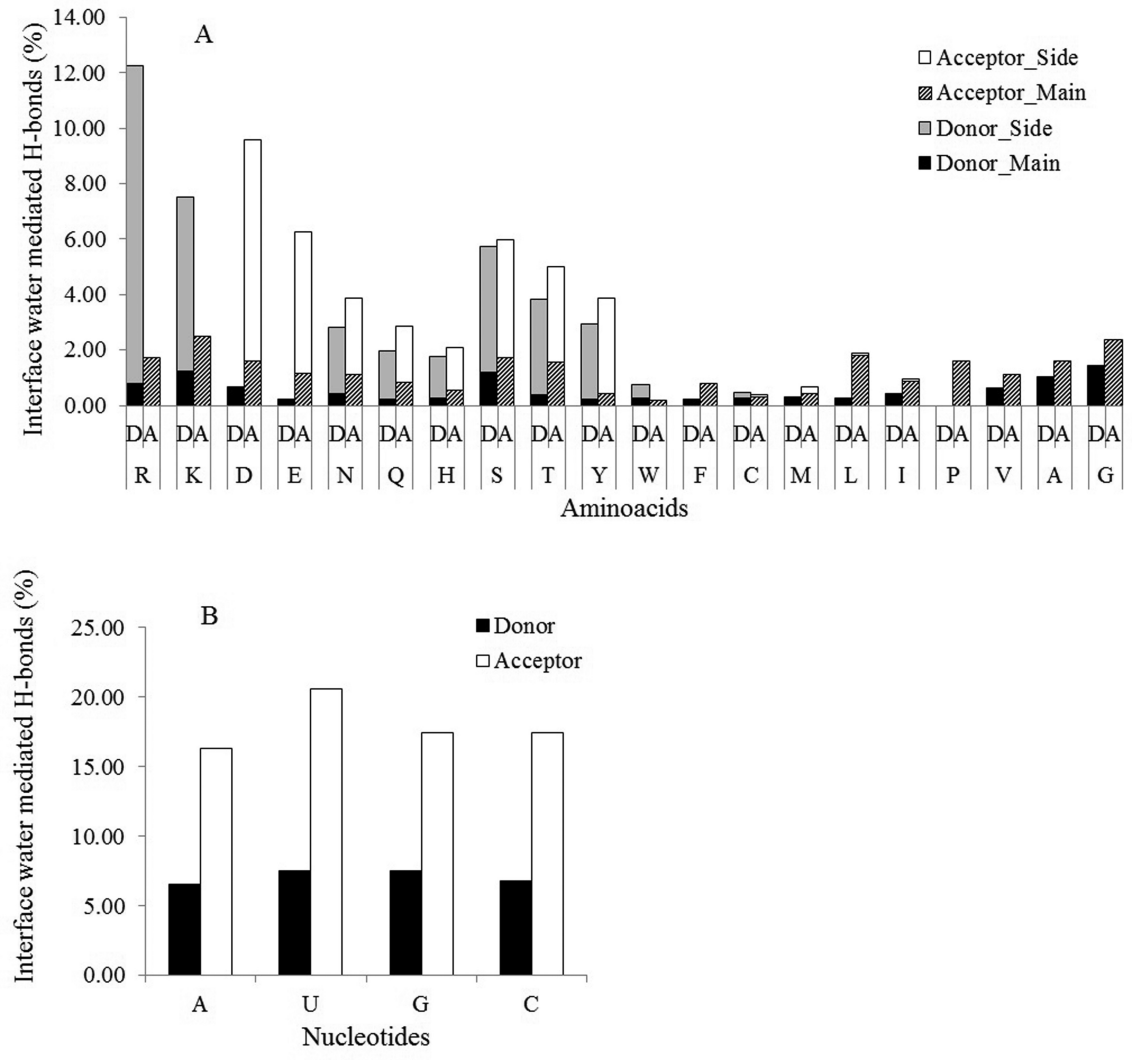
Percentage contribution of (**A**) amino acid residues, and (**B**) nucleotide bases to the interface water-mediated H-bonds. One-letter codes of the amino acid residues along with their contribution as the donor (D) or as the acceptor (A) are given along the abscissa in (A). One-letter codes of the nucleotide bases and their contribution as the donor (D) or as the acceptor (A) are given along the abscissa in (B).

Table 3 and Figure 3A describe the frequency of chemical groups involved in water-mediated H-bonds. The neutral polar side chains of amino acid residues have the highest contribution followed by the charged side chains, and the main chain oxygen and nitrogen. The side chains of the neutral polar amino acids like Asn, Gln, Thr and Tyr are preferable acceptors, while the side chains of His and Ser have no such preferences. Among the charged side chains, Arg and Lys are the major donors, while Asp and Glu are the major acceptors. In contrast to the water-mediated H-bonds, the charged side chains have the highest contribution in the direct H-bonds followed by the neutral polar side chains, and the main chain nitrogen and oxygen.

**Table 3.** Chemical composition of H-bonds

H-bonds	Protein–RNA		Protein–DNA^a^	
	Interface water-mediated	Direct	Interface water-mediated	Direct
Total number	4625	1740	8008	2585
With protein chain	1942	-	4087	-
With nucleotide chain	2683	-	3921	-
Protein chemical group (%)^b^				
Main chain O	24	12	21	3
Main chain N	10	14	10	23
Side chain groups				
Charged	31	43	32	37
Neutral	35	31	37	37
Nucleic acid chemical group (%)				
Phosphate^c^	30	36	56	70
Sugar^d^	45	31	14	6
Base	25	33	30	25
Guanine	7	10	9	12
Adenine	6	7	9	3
Cytosine	4	6	6	6
Uracil/Thymine	8	10	6	4

^a^Calculated on a protein–DNA dataset taken from (45) with resolution 2.6 Å or better, and having at least one interface water molecule.

^b^Charged side chain: N of Arg, Lys and O of Asp, Glu. Neutral side chain: N of Asn, Gln, His, Trp; O of Asn, Gln; OH of Ser, Thr, Tyr and S of Cys, Met.

^c^Phosphate includes O1P, O2P and P atoms.

^d^Oxygen atoms O2′, O3′, O4′ and O5′ are attributed to the sugar moiety.

**Figure 4.**
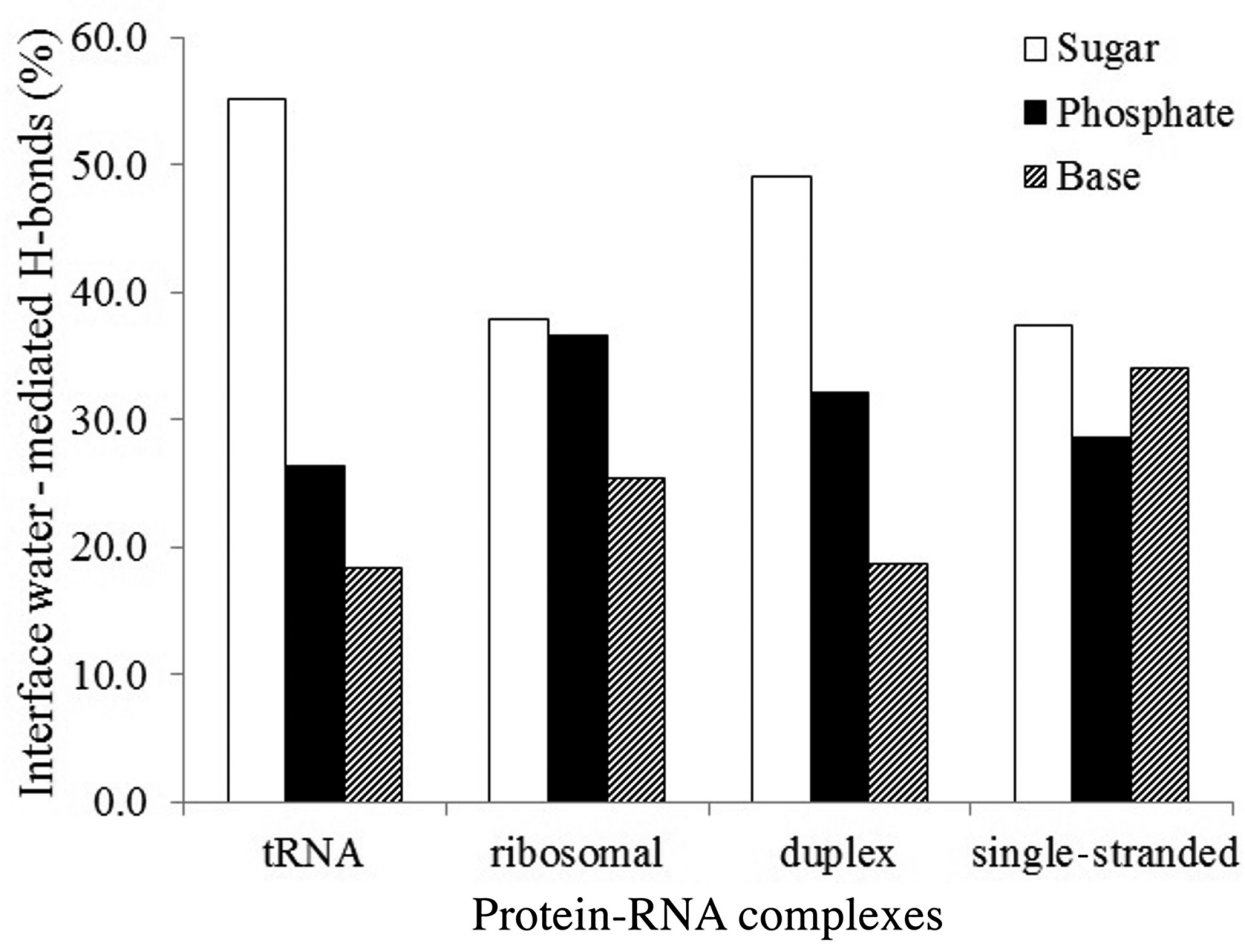
Number of interface water–RNA H-bonds in different structural classes. The phosphate includes O1P and O2P; the sugar moiety includes O2′, O3′, O4′ and O5′.

Of all the water–RNA H-bonds, the ribose contributes about 45% (14 per interface), whereas the phosphate and the bases contribute 30% (9 per interface) and 25% (7 per interface), respectively. This contribution differs in direct protein–RNA H-bonds, where the phosphate group contributes slightly more (36% or 7 H-bonds per interface) than the bases or the ribose (both contribute about 32% or 6 H-bonds per interface). Among the different bases, guanine and uracil are more frequently found in water-mediated H-bonds than adenine and cytosine. Similar trend is observed for different bases involved in direct H-bonds. Polar groups in all the four bases are frequently found as acceptors in water-mediated H-bonds rather than donors (Figure 3B). The relative contributions of ribose, phosphate and bases in water-mediated H-bonds across different classes are shown in Figure 4. It shows that the ribose contributes twice more than the phosphate and even thrice more than the bases in interfaces with tRNA. Similar trend is observed in interfaces with duplex RNA. In interfaces with ribosomal proteins, contributions of the ribose and the phosphate are almost identical, and each of them contributes more than the bases. In contrast, contributions of the sugar and the bases are almost identical, and each of them contributes more than the phosphate in interfaces with single-stranded RNA.

### Interaction with the 2′OH

The presence of the 2′OH in RNA enhances its ability to make polar interactions with the waters compared to DNA. In the present dataset, the 2′OH is involved in 31% of all water-mediated H-bonds (Table 4). This is equivalent to the percentage of phosphate–water H-bonds, and 6% more than the base–water H-bonds (Table 3). The contribution of the 2′OH in water–RNA H-bonds varies in different classes. It is involved more in interfaces with tRNA (38%) and duplex RNA (33%) compared to interfaces with ribosomal proteins (26%) and single-stranded RNA (25%). Considering all oxygen atoms within the sugar moiety, the 2′OH itself contributes 67% of all the ribose–water H-bonds. This percentage is essentially same in different classes. Of all direct protein–RNA H-bonds, the 2′OH contributes 23%, and this increases to 74% while considering only the oxygen atoms within the sugar moiety. Treger and Westhof (17) also observed the similar contribution of the 2′OH in case of direct protein–RNA H-bonds (21%). However, they worked with a smaller dataset of 45 complexes. Our analysis shows that the 2′OH is involved more in water-mediated H-bonds than in direct H-bonds. But when compared to the number of H-bonds formed by the oxygen atoms of the sugar moiety, the 2′OH is more inclined to form direct H-bonds than the water-mediated ones (Table 4).

**Table 4.** H-bonds with the 2′OH

	tRNA	ribosomal	duplex	single-stranded	Total
Interface water-mediated H-bonds					
Overall	560	153	983	987	2683
No. with ribose sugar	309	58	483	369	1219
No. with 2′OH	210	40	322	247	819
% with respect to all	38	26	33	25	31
% within sugar moiety^a^	68	69	67	67	67
Direct H-bonds					
Overall	352	80	593	715	1740
No. with ribose sugar	114	27	203	190	534
No. with 2′OH	86	23	151	134	394
% with respect to all	24	29	25	19	23
% within sugar moiety^a^	75	85	74	71	74

^a^Contribution of the 2′OH with respect to all the oxygen atoms present in the ribose sugar. Here, O3′, O4′ and O5′ oxygen atoms are attributed to the sugar moiety.

### Hydration pattern

**Figure 5.**
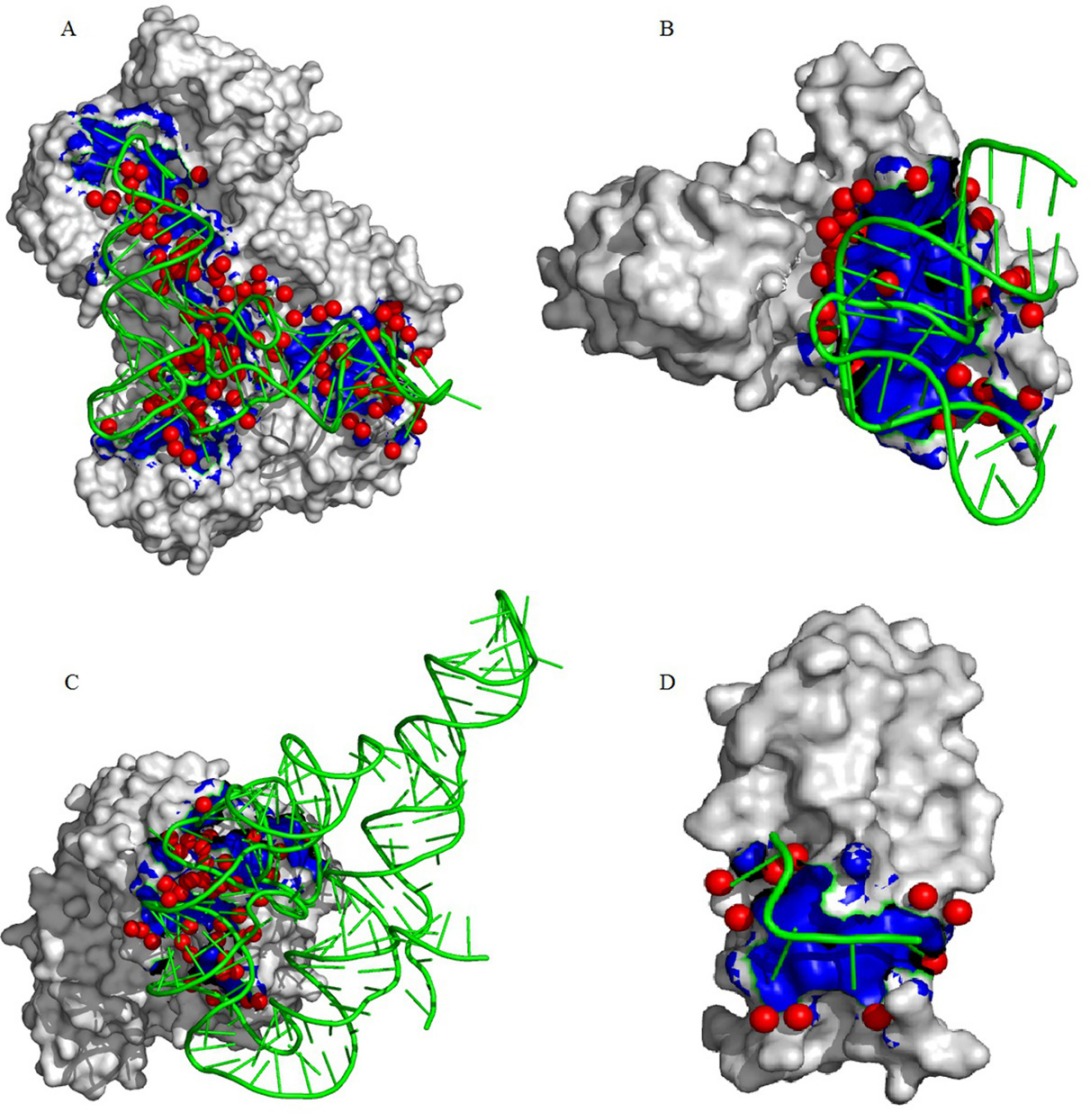
Hydration pattern in protein–RNA interfaces. In each diagram, the protein chain is shown as molecular surface and the RNA chain is shown as ribbon. The interface region is colored blue, and the interface water molecules are represented by red sphere. (**A**) A ‘wet’ interface between arginyl-tRNA synthetase and its cognate tRNA (1F7U; *d*_r_ = 0.84). (**B**) A ‘dry’ interface between ribosomal protein L1 and mRNA (2HW8; *d*_r_ = 1.18). (**C**) A ‘wet’ interface between FAB and duplex RNA (2R8S; *d*_r_ = 0.95). (**D**) A ‘dry’ interface between CspB and single-stranded RNA (3PF4; *d*_r_ = 1.46).

Depending on the spatial distribution of the interface waters, macromolecular interfaces can be distinguished into ‘wet’ and ‘dry’ categories (25). We used *d*_r_ (as described in the ‘Materials and Methods’ section) to quantify the ‘dry’ and ‘wet’ protein–RNA interfaces. Figure 5 shows the spatial distribution of the interface waters in four protein–RNA complexes. Interfaces with tRNA and duplex RNA are generally ‘wet’ with an average *d*_r_ of 0.86 and 0.98, respectively, whereas the interfaces with ribosomal proteins and single-stranded RNA are ‘dry’ with an average *d*_r_ of 1.17 and 1.07, respectively (Table 2). The interface of arginyl-tRNA synthetase and its cognate tRNA (PDB id: 1F7U) contains 116 waters. They are distributed throughout the interface, making it ‘wet’ (*d*_r_ = 0.84; Figure 5A). All the tRNA interfaces in this dataset are ‘wet’ with *d*_r_ between 0.67 and 1.00. On the other hand, all the interfaces with ribosomal proteins are ‘dry’ with *d*_r_ >1.0. This is evident in the ribosomal protein L1–mRNA interface (PDB id: 2HW8), where the waters are distributed along the periphery of the interface, making it ‘dry’ (*d*_r_ = 1.18; Figure 5B). With few exceptions, majority of the interfaces involving duplex RNA are ‘wet’ with an average *d*_r_ of 0.98. This is evident in the Fab–RNA interface (PDB id: 2R8S) where the waters are distributed throughout the interface, making it ‘wet’ (*d*_r_ = 0.95; Figure 5C). In spite of few exceptions, majority of the interfaces involving single-stranded RNA are ‘dry’ with an average *d*_r_ of 1.07. Figure 5D shows a ‘dry’ interface between cold shock protein B and single-stranded RNA (PDB id: 3PF4) where the waters are distributed along the periphery of the interface (*d*_r_ = 1.46). The distribution of the *d*_r_ values for the 89 interfaces is shown in the Supplementary Figure S4. A two-tailed *t*-test (at *α* = 0.05 level of significance) shows that there is a significant difference (*P*-value 2.34E-18) between the ‘dry’ and ‘wet’ interfaces separated by cut-off value 1.0.

### Preservation of interface water molecules

**Figure 6.**
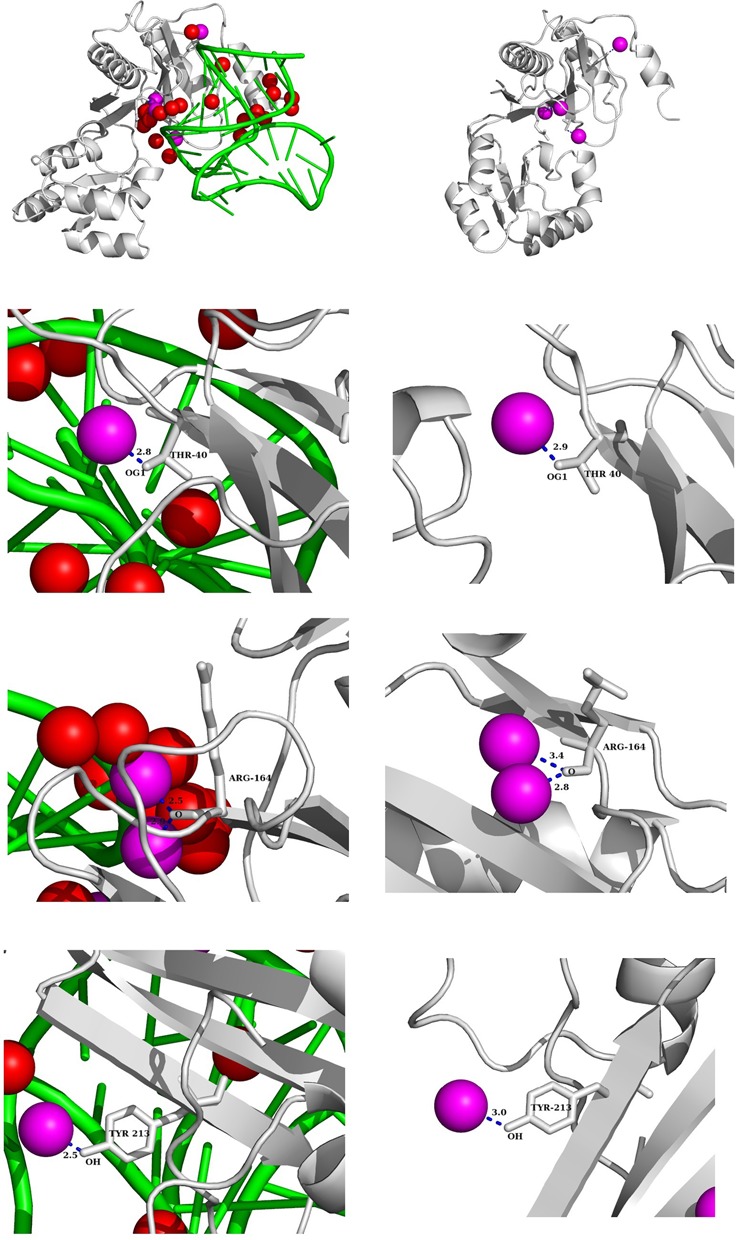
Preserved interface water molecules in a protein–RNA recognition site. In the top left panel, the bound complex between ribosomal protein L1 and mRNA (2HW8) with interface waters (in red) and preserved waters (in magenta) are shown. In the top right panel, the unbound protein and the four preserved waters are shown. Protein and RNA chains are shown in gray and green color, respectively. The panels below show the conservation of four interface waters. These preserved waters making same H-bond in the bound and unbound forms of the protein.

Preservation of waters at the protein–RNA interfaces was identified by comparing the bound structures with their corresponding unbound structures of the protein or the RNA (refer to the ‘Materials and Methods’ section). When both protein and RNA are available in the unbound form, we considered both of them to assign preserved waters. The number of preserved waters varies from 0 to 17 in the present dataset with an average of six (∼21% of the interface waters) per interface. This low average definitely under-represents the preserved waters, since in most of the cases we found either protein or RNA in the unbound form. This is exemplified by two complexes (PDB ids: 2R8S, 1JBS), where the corresponding protein and the RNA are available in the unbound form. In both the cases, the number of preserved waters increases when we considered the bound waters in the complex along with the free form of its partners. Moreover, it should be noted that the poor assignment of the waters in the bound and the unbound crystal structures results in the lack of preserved waters. We did not find any preserved water in two interfaces with single-stranded RNA: the one with TRAP (PDB id: 1C9S), and the other with ERA (PDB id: 3IEV). Although the complex TRAP-RNA is determined with high resolution (1.9 Å), it reports 86 bound waters, of which only six are at the interface. The complex between interferon-induced protein with tetratricopeptide repeats 5 (IFIT5) and a single-stranded RNA (PDB id: 4HOR) contains the highest number (17) of preserved waters. The X-ray structure of this complex reports 425 bound waters, of which 42 are at the interface. The helical domain of IFIT5 houses a positively charged cavity filled with interface waters (51). These cavity lining waters are also observed in the unbound structure of IFIT5 (PDB id: 4HOQ), and they remain preserved upon complexation by contributing to the optimization of the van der Waals interactions. Figure 6 shows preserved waters in the ribosomal protein L1–mRNA complex (PDB id: 2HW8). Here, we identified 24 interface waters, of which four are preserved. They make H-bonds with the same polar groups in the bound and the unbound form of the protein. In the entire dataset, we found 266 preserved waters, which is equivalent to 5.2 per 1000 Å^2^ of B. This density varies among the different classes: the lowest in interfaces with tRNA and the highest in interfaces with single-stranded RNA. Moreover, we found that the preserved waters make an average of 2.5 H-bonds compared to the other interface waters, which make only 1.9.

### Hydration of the protein secondary structural elements

The density of water-mediated H-bonds in different protein secondary structural elements is shown in Table 5. Here, the category ‘helices’ includes α-helix, 3_10_-Helix and π-helix; the category ‘strands’ includes residues in isolated β-strand, in extended strands or in β-ladder; and the category ‘others’ includes non-regular secondary structures. We found, about 42% of B is contributed by the helices; accordingly they are involved in 43% of all water–protein H-bonds. In contrast, the strands account for only 17% of B, and they are involved in only 20% of all water–protein H-bonds. The others account for 41% of B, and they are involved in 37% of all water–protein H-bonds. This suggests that the helices occur most frequently at the interfaces; thereby they are involved in the highest number of water-mediated H-bonds. However, when we calculated the density of water-mediated H-bonds (number per unit B), we found that, in spite of the lowest contribution to B as well as to the number of water-mediated H-bonds, the strands has the highest density, the helices has the intermediate density, and the others has the lowest density (Table 5). Nevertheless, this trend is not followed in some classes. In interfaces with tRNA, the strands has the lowest density, while in interfaces with single-stranded RNA, the helices has the lowest density. In case of direct H-bonds, the density is highest in the strands, while the helices and the others have almost similar densities, which are lower than the density in the strands.

**Table 5.** Hydration of the protein secondary structural elements

Interfaces	Water-mediated H-bonds			Direct H-bonds		
	Helices	Strands	Other	Helices	Strands	Other
tRNA	16.8	9.1	13.7	12.4	20.2	15.6
ribosomal	25.6	21.1	9.7	31.4	15.3	16.4
duplex	22.4	21.4	16.0	19.3	20.7	15.2
single-stranded	19.2	25.2	20.6	15.2	19.1	18.5
All classes	19.6	21.2	16.9	16.0	19.4	16.6

Number of H-bonds per 1000 Å^2^ of B contributed by the helices, the strands or the other (non-regular secondary structures).

### Interaction with the major and the minor grooves

The preferences of interface waters to interact with the major or the minor groove of the RNA have been analyzed in a subset containing 27 complexes with duplex RNA. The major groove contains N6/O6, N7 atoms of purines and O4, N4 atoms of pyrimidines. The minor groove contains N2, N3 atoms of purines and O2 atom of pyrimidines. The presence of the 2′OH in the backbone prevents RNA from adopting a B-form helix. Rather, the double-helical RNA resembles the A-form structure of the DNA. Unlike DNA, the minor groove in RNA is wide and shallow, and hence more accessible, whereas the major groove is so narrow and deep that it is not accessible to amino acid side chains from partner proteins. This is also true for the solvent waters as it is evident from the data in Table 6. Of all the H-bonds between the water molecules and the Watson–Crick paired bases, 40% are with the atoms in the major groove of RNA, while 60% are with the atoms in the minor groove. In protein–DNA interfaces, these values are 66 and 33% for the major and the minor grooves of DNA, respectively. This indicates that the RNA major groove is relatively less hydrated than its minor grove in protein–RNA interfaces, while the major groove of DNA is relatively more hydrated than its minor groove in protein–DNA interfaces.

**Table 6.** Hydration of the major and the minor groove

H-bond percentage	Protein–RNA^a^	Protein–DNA^b^
Major groove atoms	40	66
Minor groove atoms	60	33

Percentage of interface water-mediated H-bonds with the major and the minor groove atoms.

^a^Calculated on 27 duplex RNA (Class C).

^b^Calculated on a dataset of 115 protein–DNA complexes taken from (45).

## DISCUSSION

Water molecules are the integral part of macromolecular binding interfaces. They contribute to the optimization of the van der Waals interactions by filling up the interface cavities, leading to the maximization of the surface complementarity. Besides their structural role, they make polar interactions across the interfaces that can essentially bridge the two interacting surfaces. They also play an important role in shielding the electrostatic repulsions between the like charges at the binding site. In the present study, we aim to provide a comprehensive analysis of the role of interface water molecules in protein–RNA recognition. We have identified the bound crystallographic waters at the protein–RNA interfaces in a non-redundant dataset of 89 complexes curated from the PDB. In preparing the dataset, we limited ourselves to the binary complexes only, and kept the multisubunit assemblies like ribosomes and viral capsids for a separate study. The results obtained in this study are compared with the hydration properties of binary protein–DNA and protein–protein interfaces.

We find that the average size of a protein–RNA interface is smaller (2400 Å^2^) than the average size of a protein–DNA interface (3100 Å^2^), but larger than that of a protein–protein interface (1886 Å^2^). This is consistent with the data obtained in the previous studies (34,36,49). An average protein–RNA interface contains 28 waters, which is equivalent to a density of 12 molecules per 1000 Å^2^ of B. Similar density of waters was also observed in a smaller dataset assembled by Bahadur *et al.* (36). We find that the density varies in different classes. While the interfaces with tRNA exhibit the lowest density, the interfaces with ribosomal proteins exhibit the highest. This reflects their relative hydrophobic nature, where the interfaces with tRNA are more hydrophobic than those with ribosomal proteins (Table 1). In comparison, the density of waters in protein–RNA interfaces is lower than in protein–DNA interfaces, but higher than in protein–protein interfaces.

Interface waters are involved in polar interactions through H-bonds. We observe that the frequency of waters involved in one or two H-bonds is higher than those involved in three or four H-bonds. Moreover, we find a number of interface waters do not make any H-bond. These non-H-bonded interface waters are most often trapped at the interface cavities (50), and are within a distance of van der Waals interaction from at least one binding partner. This suggests that these waters may be involved in optimizing the van der Waals interactions, rather than making polar interactions as observed in the protein–DNA interfaces (17,27,49). Whereas the protein–protein interfaces are well packed and contain fewer waters than the protein–nucleic acids interfaces (26,27,34,52,53). We find an average protein–RNA interface makes 52 water-mediated H-bonds (∼22 per 1000 Å^2^ of B) with a biased distribution; RNA makes more than its partner protein. This is translated into one water-mediated H-bond per 47 Å^2^ of B, which is much higher than the direct H-bond density (one per 125 Å^2^ of B). This suggests that the water-mediated polar interactions play a greater role in the stability of protein–RNA interfaces than the direct H-bonds. The water-mediated H-bond density of an average protein–RNA interface is similar to that of an average protein–DNA interface, but higher than that of an average protein–protein interface. About one-third of the interface waters bridge the protein–RNA interfaces by making H-bonds with the polar groups on both sides. This value is almost similar to the observation made by Treger and Westhof (17) in a smaller dataset. We find the density of bridging waters is higher at the protein–nucleic acids interfaces than at the protein–protein interfaces.

Our study shows that the neutral polar side chains of amino acids contribute about one-third of all water–protein H-bonds in a protein–RNA interface. In comparison, the charged side chains contribute little less. This observation flipped in case of direct protein–RNA H-bonds, where the charged side chains, mainly from Arg and Lys, contribute more than the neutral polar side chains. This is in agreement with the findings made in the previous studies that the positively charged amino acid side chains play a greater role in RNA recognition (26,34,36,49). Between the main chain oxygen and nitrogen, the former prefers to make water-mediated H-bonds, while the latter prefers to make direct H-bonds. They are mainly involved in recognition of the bases as was observed by Allers and Shamoo (54).

A systematic dehydration analysis of B-DNA showed that water molecules can be easily removed from the ribose and the bases compared to the phosphate (29,55). This is also evident in our study, where the ribose is highly dehydrated compared to the bases and the phosphate in protein–DNA interfaces (Table 3). On the contrary, mainly due to the presence of the 2′OH, the ribose is less dehydrated than the phosphate and the bases in protein–RNA interfaces. The effect of the 2′OH is also observed in direct H-bonds, where the ribose plays a greater role in protein–RNA recognition than in protein–DNA recognition. Previous studies also identified the significant role of the 2′OH in direct H-bonds at protein–RNA interfaces (26,36,54). In addition, we show that the 2′OH plays a significant role in water-mediated polar interactions as well.

Polar interactions with the bases determine the sequence specificity (54,56–58). However, a handful of examples are present in our dataset where the base sequence can also be read indirectly. The direct as well as water-mediated H-bonds with the bases are highly dependent on the conformation of the nucleic acids. Unlike DNA, which generally exists in double helical form, RNA displays wider variety of conformations and shapes (extended conformation, double helix, loops and other irregular structural elements). Moreover, the flexibility of RNA is governed by six backbone torsional angles, whereas the flexibility of protein is governed by only two torsional angles. This makes RNA more flexible than protein and undergoes large conformational changes when binds to a protein (59). We also observed that the RNA undergoes large conformational changes upon binding its partner in a subset of bound and unbound structures used for identifying preserved waters. This is exemplified in Supplementary Figure S5. Here, the sarcin/ricin domain (SRD) of 23S rRNA undergoes relatively large conformational change (3.4 Å) upon binding to ribotoxin restrictocin. Here, we identified six preserved water molecules, and their H-bond with the polar groups preserved in the bound and unbound structures, even if there is a side chain orientation associated with these polar groups. In contrast to the protein–DNA interfaces, the bases at the protein–RNA interfaces are more frequently recognized by the direct H-bonds than the water-mediated ones. Among the different bases, G and U prefer to make water-mediated as well as direct H-bonds over A and C.

We find the helices contribute highest to the protein–RNA interface area followed by the non-regular secondary structures and the strands. The highest frequency of occurrence of the helices in protein–RNA interfaces was also observed by Treger and Westhof (17). Because of their highest occurrence, the helices make the highest number of water-mediated as well as direct H-bonds. However, when we calculated the density of water-mediated H-bonds per unit interface area, we find that it is highest in the strands, intermediate in the helices and lowest in the non-regular secondary structures.

The major groove of DNA is wide and easily accessible than its minor groove, so that the sequence-specific recognition of DNA helices is generally determined by the major groove interactions. This easy accessibility makes the major groove atoms participate in water-mediated interactions more frequently than the minor groove atoms (24,26,29,60). Our analysis shows that the major grove atoms make twice as much as water-mediated H-bonds than the minor groove atoms in protein–DNA interfaces. In case of RNA, the minor groove is wide and shallow, hence easily accessible for a sequence-specific binder than its major groove, which is narrow and deep. Likewise, the minor groove is relatively more involved in water-mediated interactions than the major groove (3,60,61). The above observation is justified in our analysis on a subset of interfaces involving duplex RNA, where we find the minor groove atoms are involved in higher number of water-mediated H-bonds than the major groove atoms.

It has been well studied that the dehydration of the non-polar groups upon macromolecular binding can lead to an increased binding affinity owing to the favorable gain in entropy associated with the release of well-ordered water molecules into the bulk solvent (62–64). On the other hand, dehydration of the polar groups upon binding can decrease the binding affinity (65). It has also been observed that the flexibility and the structural rearrangements associated with the antigen–antibody interactions often affect the water architecture within the interface (16,66). Reichman *et al.* (19) suggested that exposed waters within the protein–protein interface may be a good site for protein engineering, while buried or mostly buried waters should be left unchanged. In this study, we have identified the preserved water molecules that are not displaced upon binding of the two partners at the protein–RNA interfaces. This preservation indicates a non-random distribution of waters at the protein–RNA-binding site, which is often associated with the conformational changes in the binding partners upon complexation (Supplementary Figure S5). We have identified these preserved waters make higher number of H-bonds compared to the other interface waters. This is in coherence with the previous studies that showed the water molecules with higher coordinate number of H-bonds and tighter binding energy are less likely to be replaced (21,67). This establishes that the preserved waters play very important role in the binding affinity. They should be treated carefully while engineering a protein–RNA interface. Moreover, a prior knowledge about the preserved waters would help us to design drugs that target protein–RNA interfaces. Incorporation of the interface waters as well as a prior knowledge about their preservation could improve the accuracy of protein–RNA docking algorithms as well as the binding affinity prediction methods.

## CONCLUSION

Understanding protein–RNA interactions at the structural level is immensely important to apprehend the cellular processes occurring in the aqueous environment, where the water molecules invariably play an important role. We have performed a comprehensive analysis to understand the role of water molecules in protein–RNA recognition, and identified interface waters that do not displace upon complexation. Our understanding will help in drug design targeting protein–RNA interfaces, by knowing which waters are important in mediating the interaction between a protein and a RNA, and which, instead, can be targeted for displacement. Our analysis will also help to improve accuracy of protein–RNA docking methods, as well as binding affinity predictions, which are becoming the active field of interest in understanding protein–RNA interaction and designing novel protein–RNA complexes.

## SUPPLEMENTARY DATA

Supplementary Data are available at NAR Online.

SUPPLEMENTARY DATA
