# Supplementary material for: Hydration of protein–RNA recognition sites
Source: Nucleic Acids Res. 2014 Aug 11;42(15):10148–60. doi: 10.1093/nar/gku679 (PMC4150782; doi:10.1093/nar/gku679)
Supplement: SUPPLEMENTARY DATA [file supp_42_15_10148__index.html]

Hydration of protein–RNA recognition sites — Hydration of protein–RNA recognition sites — SUPPLEMENTARY DATA 

# Hydration of protein–RNA recognition sites

## SUPPLEMENTARY DATA

**Files in this Data Supplement:**

- SUPPLEMENTARY DATA
