# Supplementary material for: Hydration of protein–RNA recognition sites
Source: Nucleic Acids Res. 2014 Aug 11;42(15):10148–60. doi: 10.1093/nar/gku679 (PMC4150782; doi:10.1093/nar/gku679)

Supplementary Table S1: Dataset of protein-RNA complexes with interface water molecules.

Complex[a]				Unbound[b]		B[c] (Å^2)	IW[d]	BW[e]	HW[f]	PW[g]	d_r[h]
PDB id	Protein	RNA	Res (Å)	Protein	RNA						
Class A: Complexes with tRNA (12)											
1C0A (A:B)	*E. coli* aspartyl-tRNA synthetase	tRNA-Asp	2.4			4504	59	20	98		0.9
1F7U (A:B)	*S. cerevisiae* arginyl-tRNA synthetase	tRNA-Arg	2.2			5767	116	34	198		0.84
1FFY (A:T)	*S. aureus* isoleucyl-tRNA synthetase	tRNA-Ile	2.2			4970	55	24	123		0.82
1J1U (AA′:B)	*M. jannaschii* tyrosyl-tRNA synthetase	tRNA-Tyr	2.0			2242	37	10	47		0.94
1N78 (A:C)	*T. thermophilus* glutamyl-tRNA synthetase	tRNA-Glu	2.1	1J09:A		4510	52	16	110	12	0.93
1QTQ (A:B)	*E. coli g*lutaminyl-tRNA synthetase	tRNA-Gln	2.3	1NYL:A		5202	28	17	71	1	0.79
1U0B (B:A)	*E. coli* cysteinyl-tRNA synthetase	tRNA-Cys	2.3	1LI5:B		4557	18	11	50	1	0.89
2DLC (XX’:YY)	*S. cerevisiae* tyrosyl-tRNA synthetase	tRNA-Tyr	2.4			4265	10	3	20		0.67
2ZM5 (A:C)	*E. coli* isopentenyl pyrophosphate transferase	tRNA-Phe	2.6			3936	32	13	62		0.98
2ZUE (A:B)	*P. horikoshii* arginyl-tRNA synthetase	tRNA-Arg	2.0			4592	31	16	69		0.91
3VJR (A:B)	*E. coli* peptidyl-tRNA hydrolase	tRNA CCA acceptor	2.4	2PTH:A		1354	8	3	16	2	0.8
3ZJT (A:B)	*E. coli* leucyl-tRNA synthetase	tRNA-Leu	2.2			4322	22	12	50		0.8
Class B: Complexes with ribosomal protein (5)											
1DFU (P:MN)	*E. coli* Ribosomal protein L25	5s rRNA	1.8			1688	49	10	78		1.22
1FEU (A:BC)	*T. thermophiles* Ribosomal protein L25	5s rRNA	2.3			1595	15	5	32		1.16

1MJI (A:C)	*T. thermophilus* ribosomal protein L5	5S rRNA fragment	2.5			1745	12	4	23		1.1
1SDS (C:FF′)	50S ribosomal protein L7Ae	box H/ACA sRNA	1.8	1XBI:A		1199	24	8	39	6	1.2
2HW8 (A:B)	Ribosomal protein L1	mRNA	2.1	1AD2:A		2401	24	12	55	4	1.18
Class C: Complexes with duplex RNA (27)											
1DI2 (AB:DE)	dsRBD of RNA-binding protein A	dsRNA	1.9			1840	59	12	82		0.94
1HQ1 (A:B)	*E. coli* SRP	4.5S RNA domain IV	1.5			1364	28	10	50		1.23
1MSW (D:R)	T7 RNA polymerase	RNA transcript	2.1			1827	9	2	17		1.05
1N35 (A:BC)	Reovirus polymerase	dsRNA	2.5	1MUK:A		3244	26	5	40	9	1.12
1OOA (A:C)	NF-kappaB p50	aptamer RNA	2.5			1909	30	13	45		1.01
1R3E (A:CDE)	Pseudouridine synthase TruB	tRNA fragment	2.1		1EHZ:A(49-65)[i]	3682	33	16	65	13	1.01
1R9F (AA′:BC)	RNA silencing suppressor protein p19	siRNA	1.9			3216	23	7	60		0.83
1YVP (B:EFH)	Ro autoantigen	Y RNA	2.2	1YVR:A		4207	43	18	82	12	1
2BGG (A:PQ)	*A. fulgidus* PIWI	siRNA	2.2	1W9H:A		2240	30	11	51	4	0.98
2EZ6 (AB:CD)	RNase III	28-mer RNA	2.1	1JFZ:AB		5193	70	21	108	6	0.94
2GXB (A:EF)	Z alpha domain of adenosine deaminase	dsRNA	2.3			768	17	5	33		1.14
2HVY (ACD:E)	H/ACA RNP	H/ACA RNA	2.3			4310	15	3	21		0.7
2PJP (A:B)	*E. coli* SelB	SECIS RNA	2.3			1300	20	10	35		0.86
2QUX (AB:C)	Phage PP7 coat protein	hairpin RNA	2.4	2QUD:AB		1754	18	6	24	7	1.11
2R8S (LH:R)	synthetic FAB	ribozyme	2.0	2HFF:AB	1HR2:A	2510	44	13	116	3	0.95

2Y8W (A:B)	Endoribonuclease Cse3	hairpin RNA	1.8	1WJ9:A		3258	57	22	102	4	1.03
2YKG (A:CD)	RIG-I	dsRNA	2.5			2150	13	4	22		0.81
2ZKO (AB:CD)	NS1 protein of influenza A	A-form dsRNA	1.7	2Z0A:AB		2466	60	23	134	13	1.04
3BSN (A:PT)	Norwalk virus polymerase	RNA	1.8			3111	64	22	122		0.95
3BT7 (A:C)	Methyltransferase TrmA	T-arm analogue	2.4		1EVV:A	2230	16	9	31	4	0.93
3DD2 (LH:B)	Thrombin	26-mer RNA	1.9	1JOU:AD		1822	29	13	58	3	0.91
3EQT (AB:CD)	Helicase DHX58	dsRNA	2.0	2W4R:AA′		2705	29	11	63	5	1
3KS8 (AB:EF)	Polymerase cofactor VP35	dsRNA	2.4	3KS4:AB		1663	16	8	41	3	0.78
3OL6 (A:BCD)	Poliovirus polymerase	RNA	2.5	1RA6:A		4174	47	21	100	14	0.94
3RW6 (A:H)	Nuclear RNA export factor 1	CTE RNA	2.3			2699	20	9	53		1.17
4ATO (A:G)	*B. thuringiensis* ToxN	TOXI	2.2			2264	44	17	61		1.05
4L8H (AB:R)	Bacteriophage Qβ coat protein	operator hairpin RNA	2.4			1994	9	2	14		0.89
Class D: Complexes with single-stranded RNA (45)											
1C9S (L:W1-7)[i]	TRAP	ssRNA	1.9	2EXT:A		1027	6	3	12	0	1.13
1G2E (A:B)	RRM domain of the HuD protein	class II ARE fragment	2.3			2776	41	17	77		1.06
1JBS (A:C)	Restrictocin	SRD RNA analogue	2.0	1AQZ:A	1Q9A:A	1313	28	7	37	15	0.79
1JID (A:B)	*H. sapiens* SRP19	SRP RNA	1.8		1D4R:A	1436	40	10	58	3	1.06
1K8W (A:B)	Pseudouridine synthase B	T stem-loop RNA	1.9			2973	33	24	91		1.03
1KNZ (AB:W)	Rotavirus NSP3	mRNA	2.5			1920	14	6	26		1.04

1LNG (A:B)	*M. jannaschii* SRP19	7S SRP RNA	2.3		1Z43:A	2367	27	13	57	4	1.05
1M5O (C:B)	U1 Snp	Hairpin ribozyme	2.2	1OIA:A		1766	28	8	38	2	1.03
1M8W (A:CE)	Pumilio-homology domain	Nre1-19 RNA	2.2	1M8Z:A		2110	19	5	32	6	0.91
1UVI (A:D)	phi6 RNA polymerase	6-mer RNA	2.2	1HHS:A		1814	2	1	5	2	1.18
1WPU (A:C)	HutP antitermination protein	hut mRNA	1.5	1WPV:A		1357	15	3	21	4	1.16
1WSU (A:E)	Elongation factor SelB	SECIS RNA	2.3	1LVA:A			14	7	25	2	1.18
1ZH5 (AB:C)	La autoantigen	3’-terminii mRNA	1.9			1849	37	12	48		1.03
2ANR (A:B)	Nova-1 KH1/KH2 domain	hairpin RNA	1.9			1220	8	0	9		1.24
2ASB (A:B)	Nus A	rRNA	1.5	1K0R:A		2316	36	8	61	9	1.13
2B3J (AB:E)	Bacterial tRNA adenosine deaminase	Anticodon stem-loop of tRNA-Arg2	2.0			2098	21	8	41		0.92
2BH2 (A:C)	Methyltransferase RumA	23S rRNA	2.2	1UWV:A		4491	32	13	66	10	0.93
2DB3 (A:E)	DEAD-box helicase vasa	ssRNA	2.2			1193	22	5	27		1.05
2G4B (A:B)	Splicing factor U2AF	Polypyrimidine	2.5			1161	4	3	13		1.29
2I82 (A:E)	Pseudouridine synthase RluA	Anticodon stem-loop of tRNA-Phe	2.1			3018	16	13	51		0.8
2J0S (ACDT:E)	Exon junction complex	mRNA	2.2			1436	11	4	18		1.07
2JEA (AB:C)	*S. solfataricus* exosome	substrate RNA	2.3	2JE6:AB		1533	7	4	19	4	0.61
2JLU (A:C)	Serine protease subunit NS3	ssRNA	2.0	2JLQ:A		1925	22	8	43	8	0.91
2PY9 (B:E)	Poly(rC)-binding protein 2	human telomeric RNA	2.6			1060	6	2	11		1.29
2Q66 (A:X)	*S. cerevisiae* poly(A) polymerase	polyA	1.8	2HHP:A		1811	35	13	56	9	1.07

2VNU (D:B)	Exonuclease Rrp44	ssRNA	2.3			3166	37	14	72		0.91
2XNR (A:C)	Nab3-RRM	UCUU recognition sequence	1.6	2XNQ:A		926	4	2	7	1	1.57
2XS2 (A:B)	RRM from murine DAZL	3’-UTRs of mRNA	1.4			1298	33	10	43		1.16
2XZO (A:D)	Upf1 helicase	polyU	2.4			2006	14	7	35		1.05
3D2S (A:E)	MBNL1 ZnF3/4	pre-mRNA	1.7	3D2Q:A		569	6	4	14	3	1.17
3I5X (A:B)	Mss116p	polyU	1.9			2228	19	6	44		0.95
3IEV (A:D)	GTPase era	16S rRNA	1.9		1SDR:B	2273	21	8	37	0	0.99
3K5Q (A:B)	FBF	mRNA	2.2			2597	33	14	68		0.98
3M7N (DG:Y)	Exosome complex exonuclease	Bacterial RNA	2.4			1538	10	8	26		0.94
3MDG (AB:C)	CFI(m)25	pre-mRNA	2.2	3BAP:AA′		1069	13	4	21	4	1.26
3NMR (A:B)	CUG-binding protein 1	UGU-rich mRNA	1.9			1096	14	6	30		1.35
3O8C (A:C)	HCV NS3 helicase	polyU	2.0	3O8B:A		1909	32	13	56	13	1.06
3PF4 (AB:R)	CspB	ssRNA	1.4	1CSP:A		963	11	4	18	1	1.46
3R2C (AJ:R)	NusB-NusE	BoxA RNA	1.9			2272	14	3	20		0.97
3T5N (A:C)	Lassa virus nucleoprotein	ssRNA	1.8			1910	21	10	39		0.96
4H5P (AB:E)	Nucleocapsid	polyU	2.2	3LYF:AB		4482	51	26	130	16	1.08
4HOR (A:X)	IFIT	polyC	1.9	4HOQ:A		1747	42	13	78	17	1.12
4J7M (A:B)	Dom3Z	polyU	1.7			1268	25	11	55		0.99
4M59	Chloroplast ppr10	psaJ intergenic region	2.5			7842	16	12	50		1.02

(AB:CD)											
4MDX (AB:C)	mRNA interferase MazF	mRNA	1.5	1NE8:AA′		2127	39	10	69	7	1.22

[a]Four-letter PDB code of protein-RNA complexes used in the dataset with the respective chain ID(s) of the protein and the RNA molecules, which are separated by semicolon in the parentheses. Symmetry related chains are primed (e.g., AA′ in 1J1U).

[b]The unbound form of the available partners.

[c]Surface area buried between protein and RNA upon complexation.

[d]Number of interface water molecules.

[e]Number of bridging water molecules.

[f]Number of water-mediated H-bond.

[g]Number of preserved interface water molecules.

[h]Structural parameter that quantify the ‘wet’ and ‘dry’ interface.

[i]Nucleotides 49-65 are taken in RNA chain of 1EHZ, and 1 to 7 are taken in RNA chain of 1C9S.

Supplementary Table S2: List of modified amino acid residues and nucleotide bases present in the protein-RNA complexes.

UBA[a]	MBA[b]	Name	CPX[c]	UBP[d]	UBR[e]
1MA	A	6-HYDRO-1-METHYLADENOSINE-5'-MONOPHOSPHATE	1F7U,2DLC		1EHZ, 1EVV
1MG	G	1N-METHYLGUANOSINE-5'-MONOPHOSPHATE	1F7U		
2MG	G	2N-METHYLGUANOSINE-5'-MONOPHOSPHATE	1F7U,2DLC		1EHZ, 1EVV
4SU	U	4-THIOURIDINE-5'-MONOPHOSPHATE	1C0A		
5BU	U	5-BROMO-URIDINE-5'-MONOPHOSPHATE	1JID,2ANR,3O8C		
5MC	C	5-METHYLCYTIDINE-5'-MONOPHOSPHATE	1F7U,2DLC		1EHZ, 1EVV
5MU	U	5-METHYLURIDINE 5'-MONOPHOSPHATE	1C0A,1F7U,2DLC,3BT7		
5MU	U	5-METHYLURIDINE 5'-MONOPHOSPHATE	1C0A,1F7U,2DLC,3BT7		1EHZ, 1EVV
6IA	A	N6-ISOPENTENYL-ADENOSINE-5'-MONOPHOSPHATE	2DLC		
A23	A	ADENOSINE-5'-PHOSPHATE-2',3'-CYCLIC PHOSPHATE	4ATO,1JID		1D4R
A2M	A	2'-O-METHYLADENOSINE 5'-(DIHYDROGEN PHOSPHATE)	1JBS		
CCC	C	CYTIDINE-5'-PHOSPHATE-2',3'-CYCLIC PHOSPHATE	1HQ1,3RW6		
CFL	C	4-AMINO-1-(2-DEOXY-2-FLUORO-5-O-PHOSPHONO-BETA-D-ARABINOFURANOSYL)PYRIMIDIN-2(1H)-ONE 2'-FLUORO-2'-DEOXY-CYTIDINE-5'-MONOPHOSPHATE	3DD2		
CTP	C	CYTIDINE-5'-TRIPHOSPHATE	4HOR		
DG	G	2'-DEOXYGUANOSINE-5'-MONOPHOSPHATE	2Y8W		
DU	U	2'-DEOXYURIDINE-5'-MONOPHOSPHATE	2GXB		
FHU	U	(5S,6R)-5-FLUORO-6-HYDROXY-	1R3E,1K8W		

		PSEUDOURIDINE-5'-MONOPHOSPHATE			
FMU	U	5-FLUORO-5-METHYLURIDINE-5'-MONOPHOSPHATE	2BH2		
FOU	U	(5S,6R)-5-FLUORO-6-HYDROXYDIHYDROPYRIMIDINE-2,4(1H,3H)-DIONE	2I82		
G7M	G	N7-METHYL-GUANOSINE-5'-MONOPHOSPHATE	1C0A		
GDP	G	GUANOSINE-5'-DIPHOSPHATE			1D4R
GTP	G	GUANOSINE-5'-TRIPHOSPHATE	3RW6		
H2U	U	5,6-DIHYDROURIDINE-5'-MONOPHOSPHATE	1C0A,1F7U		1EHZ, 1EVV
IU	U	5-IODOURIDINE-5'-MONOPHOSPHATE	1YVP		
M2G	G	N2-DIMETHYLGUANOSINE-5'-MONOPHOSPHATE	1F7U,2DLC		1EHZ
N5M	C	5-NITROCYTIDINE 5'-(DIHYDROGEN PHOSPHATE)	3BSN		
OMC	C	O2'-METHYLYCYTIDINE-5'-MONOPHOSPHATE	1JBS		1EHZ, 1EVV
OMG	G	O2'-METHYLGUANOSINE-5'-MONOPHOSPHATE	2DLC,1JBS		1EHZ, 1EVV
OMU	U	O2'-METHYLURIDINE 5'-MONOPHOSPHATE	1JBS		
P5P	A	PURINE RIBOSIDE-5'-MONOPHOSPHATE	2B3J		
PSU	U	PSEUDOURIDINE-5'-MONOPHOSPHATE	1C0A,1F7U,2DLC		1EHZ, 1EVV
QUO	G	2-AMINO-7-DEAZA-(2",3"-DIHYDROXY-CYCLOPENTYLAMINO)-GUANOSINE-5'-MONOPHOSPHATE	1C0A		
U37	U	URIDINE 5'-5'-MONOTHIOPHOSPHATE	4J7M		
UFT	U	2'-DEOXY-2'-FLUOROURIDINE 5'-(DIHYDROGEN PHOSPHATE)	3DD2		
CAF	CYS	S-DIMETHYLARSINOYL-CYSTEINE		2XNQ	

CAS	CYS	S-(DIMETHYLARSENIC)CYSTEINE		1RA6	
MSE	MET	SELENOMETHIONINE	1MJI, 1R9F,1ZH5,2ANR, 2HW8,2I82,2PY9,2VNU, 2ZM5,3NMR		

[a]Unusual bases or amino acid residues.

[b]Corresponding bases or amino acid residues.

[c]List of protein-RNA complexes where the modified bases and residues are present.

[d]List of unbound protein structures where the modified residues are present.

[e]List of unbound RNA structures where the modified bases are present.

Supplementary Figures.

Figure S1. The number of interface water (IW), bridging water (BW), interface water mediated H-bonds (HW) and preserved interface waters (PW) at the different cut-off distances. The numbers IW and HW increases with the increase of the cut-off value used for the selection of the interface waters. However, BW and to some extent PW remain unchanged beyond 3.5 Å.

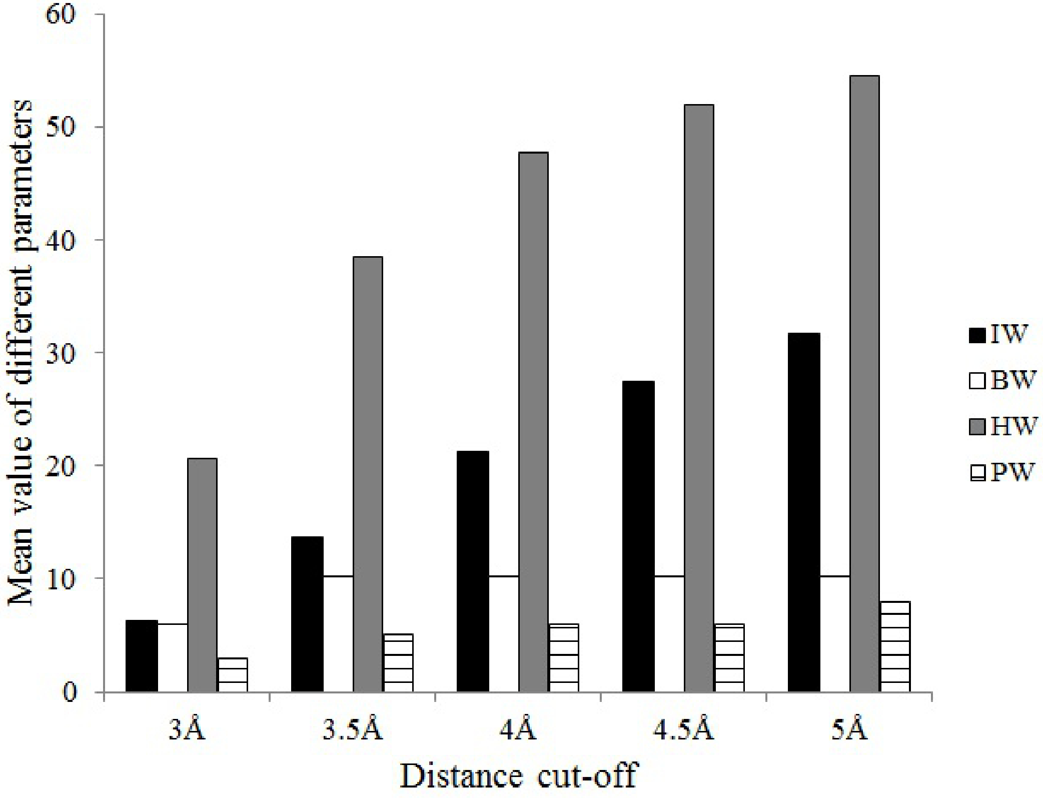

Figure S2. The distance distribution of the H-bonded and the non H-bonded interface waters from the closest interface atom. The average distance of the H-bonded waters from the closest interface atoms is 2.9 Å, whereas it is 3.5 Å for the non H-bonded waters. About 90% (68 out of 75 waters) of all the non H-bonded waters are within 3.9 Å from the closest interface atom.

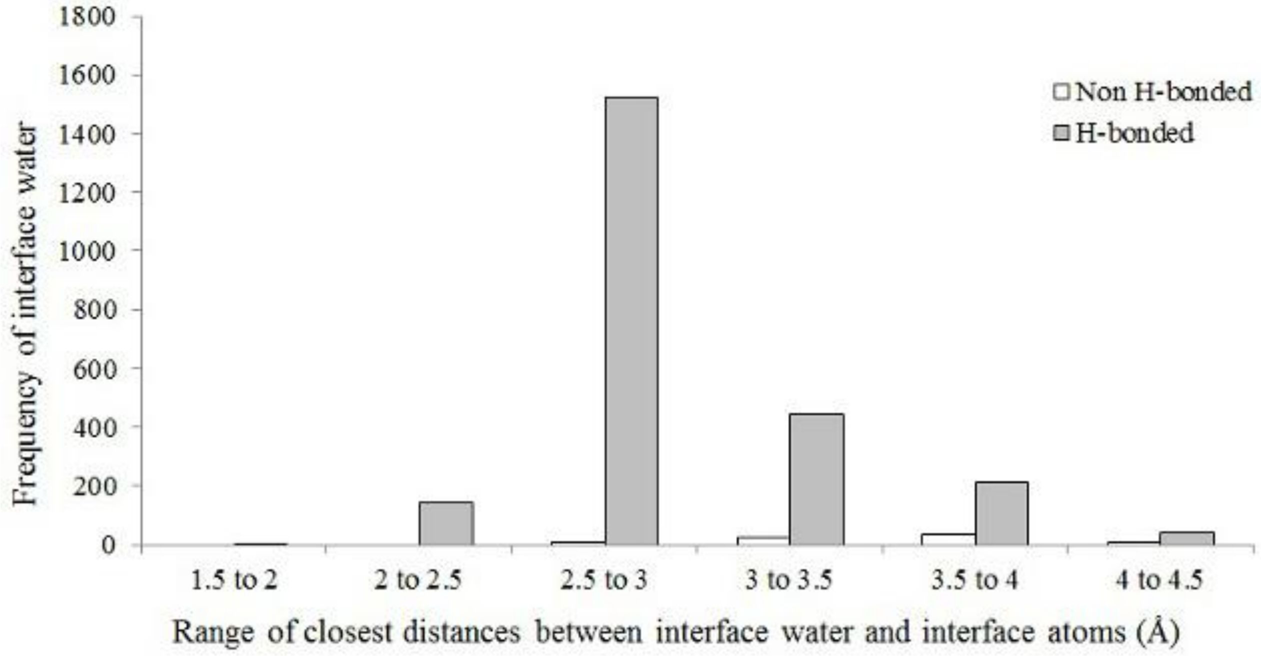

Figure S3. Interface waters do not making any H-bond with the protein or the RNA. The four interface waters in aspartyl-tRNA synthetase complexed with tRNA (PDB id: 1C0A) do not make any H-bond with either protein or RNA are represented by spheres. However, three of them (shown in magenta) are involved in H-bond with other interface waters. Only one of them (shown in red) does not participate in any H-bond. This water is buried at the interface cavity and makes van der Waals interactions with the protein side of the interface (its nearest distance from a protein atom is 3.3 Å). The protein is shown in grey surface, and the RNA is shown in green cartoon.

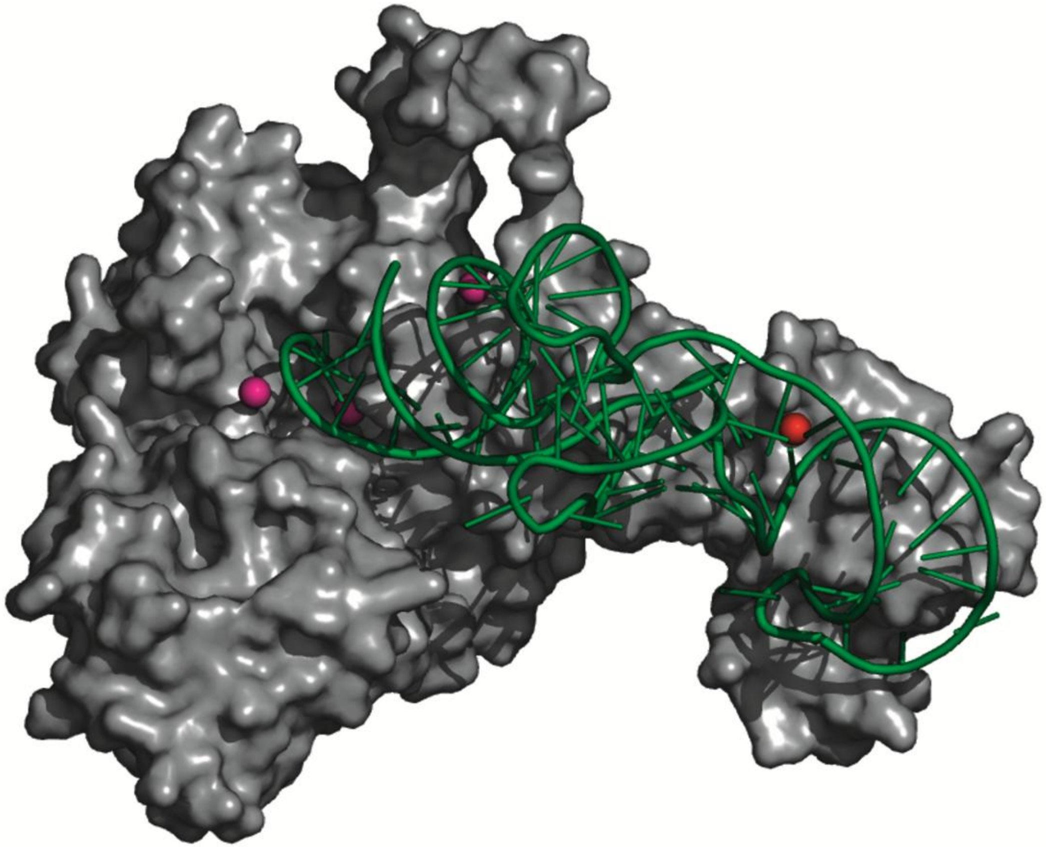

Figure S4. The distribution of d_r values of protein-RNA interfaces.

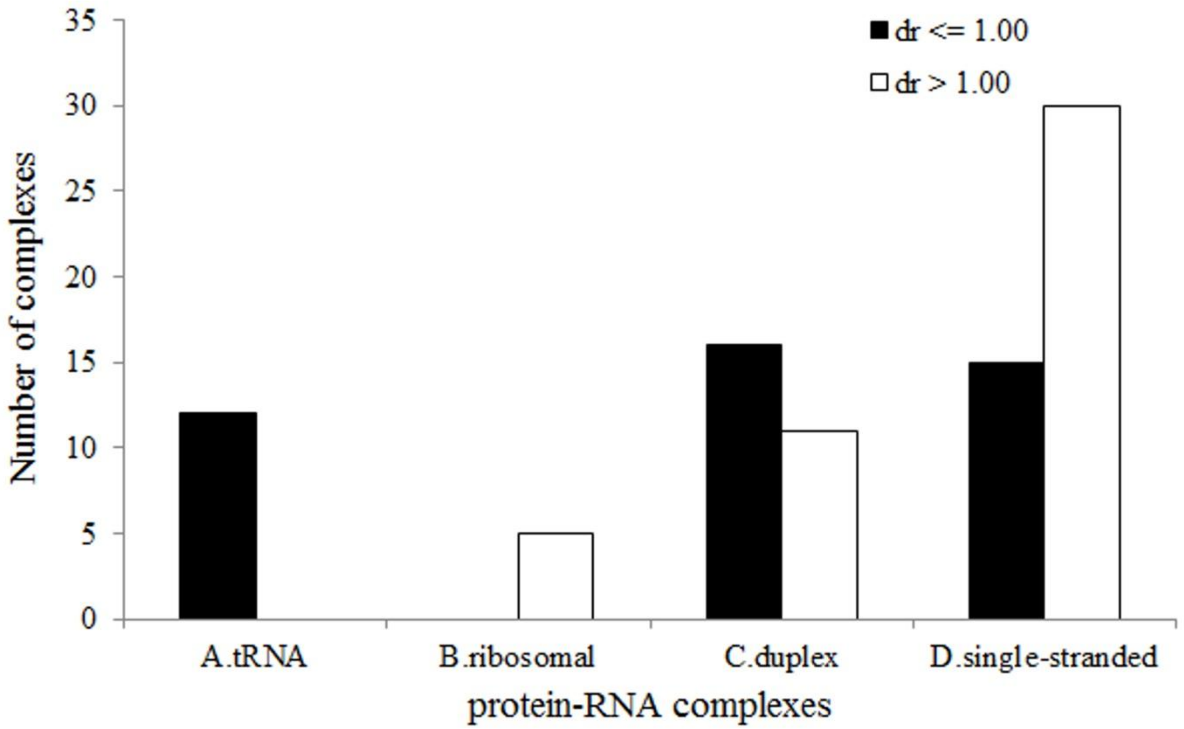

Figure S5. Preserved interface waters in superimposed bound and unbound structures of ribotoxin restrictocin complexed with SRD RNA. In the left panel, the protein component in the bound complex (PDB id: 1JBS; coloured green) is superimposed with its unbound form (PDB id: 1AQZ; coloured cyan) and the RNA component in the complex (coloured green) is superimposed with its unbound form (PDB id: 1Q9A; coloured blue). The preserved water, shown in the top panel, makes one H-bond with the OE1 of Glu95 of restrictocin in the bound as well as in the unbound form, where the side chain of Glu95 remains almost at the same position. On the other hand, the preserved water, shown in the panel below, still make the same H-bond with the N1 of C17 in the bound and unbound form of the SRD RNA, although it undergoes a large conformational change (3.4 Å) upon binding with the protein.

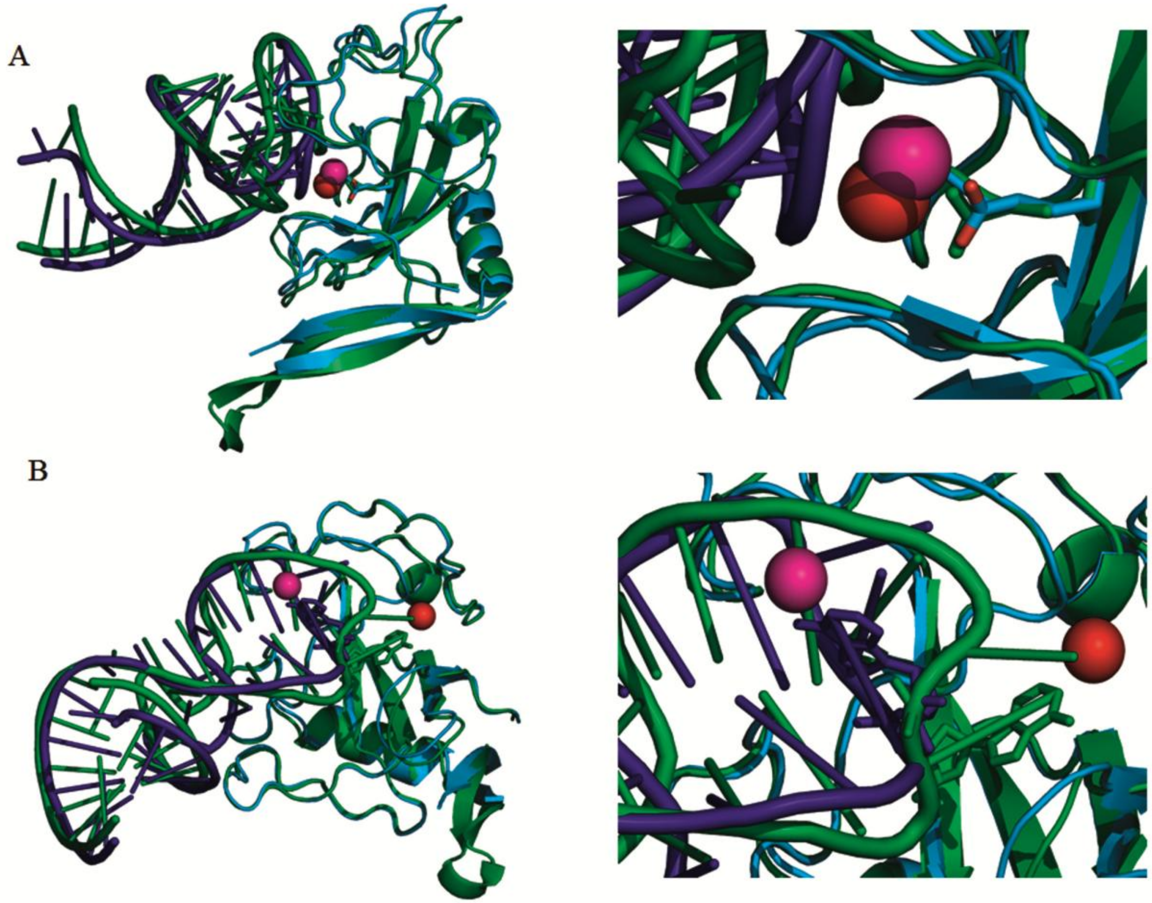

Supplement: SUPPLEMENTARY DATA [file supp_gku679_nar-00032-r-2014-File008.pdf]
